# Development of HuMiChip for Functional Profiling of Human Microbiomes

**DOI:** 10.1371/journal.pone.0090546

**Published:** 2014-03-04

**Authors:** Qichao Tu, Zhili He, Yan Li, Yanfei Chen, Ye Deng, Lu Lin, Christopher L. Hemme, Tong Yuan, Joy D. Van Nostrand, Liyou Wu, Xuedong Zhou, Wenyuan Shi, Lanjuan Li, Jian Xu, Jizhong Zhou

**Affiliations:** 1 Department of Microbiology and Plant Biology, Institute for Environmental Genomics, University of Oklahoma, Norman, Oklahoma, United States of America; 2 State Key Laboratory of Oral Diseases, West China Hospital of Stomatology, Sichuan University, Chengdu, China; 3 State Key Laboratory for Diagnosis and Treatment of Infectious Disease, The First Affiliated Hospital, Zhejiang University, Hangzhou, China; 4 Chinese Academy of Sciences, Qingdao Institute of Bioenergy and Bioprocess Technology, Qingdao, Shandong, China; 5 UCLA School of Dentistry, University of California Los Angeles, Los Angeles, California, United States of America; 6 Earth Science Division, Lawrence Berkeley National Laboratory, Berkeley, California, United States of America; 7 State Key Joint Laboratory of Environment Simulation and Pollution Control, School of Environment, Tsinghua University, Beijing, China; Auburn University, United States of America

## Abstract

Understanding the diversity, composition, structure, function, and dynamics of human microbiomes in individual human hosts is crucial to reveal human-microbial interactions, especially for patients with microbially mediated disorders, but challenging due to the high diversity of the human microbiome. Here we have developed a functional gene-based microarray for profiling human microbiomes (HuMiChip) with 36,802 probes targeting 50,007 protein coding sequences for 139 key functional gene families. Computational evaluation suggested all probes included are highly specific to their target sequences. HuMiChip was used to analyze human oral and gut microbiomes, showing significantly different functional gene profiles between oral and gut microbiome. Obvious shifts of microbial functional structure and composition were observed for both patients with dental caries and periodontitis from moderate to advanced stages, suggesting a progressive change of microbial communities in response to the diseases. Consistent gene family profiles were observed by both HuMiChip and next generation sequencing technologies. Additionally, HuMiChip was able to detect gene families at as low as 0.001% relative abundance. The results indicate that the developed HuMiChip is a useful and effective tool for functional profiling of human microbiomes.

## Introduction

Extensive studies have shown that the human microbiome plays extremely important roles in human health, nutrition, disease, and antibiotic resistance [1], [2], [3], [4], [5]. Many human disorders, such as dental caries, periodontitis, type 2 diabetes, and obesity, are closely related with changed microbial communities in the human body [3], [6], [7], [8], [9], [10], [11], [12]. Thus understanding the diversity, composition, structure, function, and dynamics of human microbiomes in individual human hosts is crucial to reveal human-microbial interactions, especially for patients with microbially mediated disorders, but challenging due to the high diversity of the human microbiome. For example, the number of microbial cells is at least ten times more than human cells in the individual human body [13], [14], and the number of microbial genes is 100 times more than their host. Although thousands of microbial species from the human body have been isolated and sequenced, especially by the Human Microbiome Project (HMP) [15], characterizing and linking the function of microbial communities to their host’s health status (e.g., obesity, liver diseases, periodontitis) is still challenging.

Microbial ecological microarrays are a technology that can be used for highly parallel detection of complex microbial communities in many environments [16], [17]. So far, a variety of microarrays, such as GeoChip, PhyloChip, HITChip, HuGChip, as well as a series of other 16S rRNA based microarrays have been developed and widely used for functional and phylogenetic profiling of microbial communities from different habitats [18], [19], [20], [21], [22], [23]. However, these microbial ecological microarrays mainly target functional genes that play important roles in biogeochemical processes in the natural environment or 16S rRNA genes, but not functional genes specifically important to the human body. Intriguingly, recent metagenomic studies suggested that a functional rather than a taxonomic core might be present within a given niche of the human microbiome, and that changes in these cores might lead to different physiological states [5], [11], [24], [25].

In this study, we aimed to develop a functional gene based microarray to target key microbial functional processes related with human health, disease and nutrition. The developed HuMiChip was applied to characterize the human microbiome with human gut and oral samples. Also, we compared the functional gene profiles of human gut and oral samples obtained by the HuMiChip and by next generation sequencing technologies, and consistent results were observed. This study demonstrates that the developed HuMiChip is a useful and effective tool for functional profiling of human microbiomes.

## Materials and Methods

### Sequence retrieval, probe designing and microarray synthesis

The HuMiChip was developed using a pipeline (Figure S1) modified from the GeoChip 3.0 and 4.0 design [26]. Reference protein sequences for each selected gene family were retrieved from the KEGG database and subject to multiple sequence alignment, and an HMM model was built using the HMMER program [27]. A total of 322 bacterial genome sequences and 31 shotgun metagenomes [24] were downloaded: 300 from NCBI database, 16 from HOMD [28], 6 from Oralgen database [29], and 31 human gut metagenomes from MG-RAST server [24], [30], [31], which formed a Mother database (MotherDB). Protein sequences were extracted and searched against the pre-built HMM models from reference sequences collected from the KEGG database [32]. Corresponding nucleotide sequences were extracted and subject to probe design by CommOligo 2.0 [33] using probe design criteria described previously [26]. Candidate probes were searched against the whole MotherDB for specificity. The best probes were selected for microarray fabrication by Roche NimbleGen (Madison, WI).

### Sampling, DNA extraction, purification and quantification

Oral subgingival/supragingival and fecal samples were collected from subjects at the West China Hospital of Stomatology, Sichuan University (oral samples) and the First Affiliated Hospital of Zhejiang University (fecal samples), respectively. A total of 86 individuals were recruited for sample collection, among which 62 were oral samples representing five groups of oral microbiota, and 24 were fecal samples representing gut microbiota. Subgingival plaque was collected for periodontitis patients, subgingival and supragingival plaque from teeth #11-18 and #31-38 was collected for healthy individuals, and supragingival plaque from teeth #11-18 and #31-38 was collected for patients with dental caries. All patients were provided written informed consent and research was approved by the local (the West China Hospital of Stomatology of Sichuan University and the First Affiliated Hospital of Zhejiang University) ethics committee and Institutional Review Broad (IRB), respectively.

The following criteria were applied to identify healthy individuals and patients with moderate/severe dental caries and moderate/advanced periodontitis. General criteria for patients with periodontitis/dental caries were [34]: (i) aged between 20 and 70 years; (ii) medically healthy; (iii) no previous periodontal/dental caries treatment and no antibiotic use within the past 6 months; and (iv) willing to consent to the clinical examination and microbial sampling. Moderate periodontitis was identified with 4 mm < probe depth (PD) ≤ 6 mm,attachment loss (AL) 3∼5 mm, 1/3 root length < alveolar bone destruction (ABD) < ½ root length. And advanced periodontitis was identified with PD ≥ 6 mm, AL > 5 mm, and ABD > 1/2 root length [35]. For patients with dental caries, the decayed, missing and filled tooth (DMFT) index was used to define different levels of conditions. Moderate caries was identified for patients with 0 < DMFT < 5, and severe dental caries was defined with DMFT ≥ 5. All healthy individuals must have (i) no pockets and clinical attachment loss (CAL); (ii) no alveolar bone absorption on X-ray examination; and (iii) less than 15% of sites with bleeding on probing (BOP) or redness.

For oral microbiome sampling, bacteria were separated from the paper-points by vortexing. The paper points were discarded and community DNA was extracted using the QIAamp ™ DNA micro Kit (QIAGEN Sciences, Maryland, USA) following the manufacturer’s instructions and adding a lysozyme (3 mg/mL, 1.5 h) treatment step.

For gut microbiome sampling, all fecal samples were immediately frozen on collection and stored at −70°C before analysis. A frozen aliquot (200 mg) of each fecal sample was added to a 2.0-ml screwcap vial containing 300 mg glass beads of 0.1 mm diameter (Sigma, St. Louis, MO, USA), and kept on ice until the addition of 1.4-ml ASL buffer from the QIAamp DNA Stool Mini Kit (Qiagen, Valencia, CA, USA). Samples were immediately subjected to beadbeating (45 s, speed 6.5) using a FastPrep machine (Bio 101, Morgan Irvine, CA, USA), prior to the initial incubation for heat and chemical lysis at 95°C for 5 minutes. Subsequent steps of DNA extraction followed the QIAamp kit protocol for pathogen detection.

DNA quality was evaluated by the absorbance ratios at *A*260/*A*280 and *A*260/*A*230 using spectrophotometry (NanoDrop 1000, Thermo Scientific) and final DNA concentrations were quantified with the Pico-Green kit (Invitrogen, Carlsbad, CA, USA). Only DNA samples with *A*260/*A*280 > 1.7 and *A*260/*A*230 > 1.8 were used. The extracted whole community DNA for each sample was then shipped to the University of Oklahoma (OU) for HuMiChip analysis. Since only DNA samples were used at OU, the OU IRB ruled this as non-human research so that IRB approval was not needed from OU.

### Target labeling and hybridization

The purified DNA was labeled with Cy-3 using random primers and the Klenow fragment of DNA polymerase I [36]. Labeled DNA was purified using the QIA quick purification kit (Qiagen, Valencia, CA) according to the manufacturer’s instructions, measured on a NanoDrop ND-1000 spectrophotometer (NanoDrop Technologies Inc., Wilmington, DE), and then dried down in a SpeedVac (ThermoSavant, Milford, MA) at 45°C for 45 min. Dried DNA was rehydrated with 2.68 µL sample tracking control (NimbleGen, Madison, WI, USA) to confirm sample identity. The samples were incubated at 50°C for 5 min, vortexed for 30 sec, and then centrifuged to collect all liquid at the bottom of the tube. Hybridization buffer (7.32 µL), containing 40% formamide, 25% SSC, 1% SDS, 2.38% Cy3-labeled alignment oligo (NimbleGen) and 2.8% Cy5-labeled CORS target, was added. The samples were then mixed by vortexing, spun down, incubated at 95°C for 5 min, and maintained at 42°C until hybridization. An HX12 mixer (NimbleGen) was placed onto the array using NimbleGen’s precision mixer alignment tool, and then the array was preheated to 42°C on a hybridization station (MAUI, BioMicro Systems, Salt Lake City, UT, USA) for at least 5 min. Samples (6.8 µL) were then loaded onto the array surface and hybridized approximately 16 h with mixing.

### Imaging, and data preprocessing

After hybridization, arrays were scanned at full laser power and 100% PMT gain with a NimbleGen MS 200 Microarray Scanner (Roche NimbleGen). Scanned images were gridded by NimbleScan software using the gridding file containing HuMiChip probes and NimbleGen control probes to obtain the signal intensity for each probe. Probe spots with coefficient of variance (CV) greater than 0.8 were removed. Probes with SNR (signal-to-noise ratio) less than 2 and signal intensities less than 1000 were also removed. Microarray data was then normalized based on the total signal intensity of CORS probes. Both raw and normalized data is available under NCBI GEO accession number GSE54290.

### Statistical analysis

Three different non-parametric multivariate analysis methods, adonis (permutational multivariate analysis of variance using distance matrices), anosim (analysis of similarities) and MRPP (multi-response permutation procedure), as well as detrended correspondence analysis (DCA), were used to measure the overall differences of the community functional gene structure between treatment and control samples [37]. The significance of relative abundance differences between control and treatment samples for functional gene categories was evaluated by the response ratio analysis.

### Comparative analysis of functional gene profiles by HuMiChip and NGS technologies

Gene family abundance datasets by NGS technologies were downloaded from http://www.hmpdacc.org/HMMRC/, and profiles targeting human stool and subgingival plaque samples were extracted and analyzed. The human gut and healthy human oral microbial gene family profiles by HuMiChip were extracted and compared with that by NGS technologies. Pearson correlation coefficient was calculated to estimate the correlation between the HuMiChip signal intensity and NGS relative abundance.

## Results

### Functional gene families included in HuMiChip

To monitor the functional diversity, composition, structure, and dynamics of human microbiomes, we selected 139 functional gene families that play important roles in multiple pathways. A detailed list and description of selected functional genes can be found in the supplementary information (Table S1).

#### (i) Amino acid metabolism and biosynthesis

Amino acids play central roles in building protein blocks and intermediates in metabolism. In the human body, 8 of 20 basic amino acids are essential but cannot be self-produced, and for the other 12 amino acids, 8 are conditionally essential [38]. Essential and conditionally essential amino acids must be taken from external sources, such as food and/or microbial synthesis [39]. The human gut microbiome is enriched with genes involved in the synthesis of essential amino acids [40]. Here we selected 59 gene families involved in amino acid and/or precursor synthesis, transport and metabolism in human microbiota. These gene families were selected for their important roles in degradation, biosynthesis, and conversion of essential amino acids, which are of great importance for human nutrition. Among these, 16 gene families were selected for their important roles in arginine and proline metabolism, 9 in alanine, aspartate and glutamate metabolism, 8 in phenylalanine, tyrosine and tryptophan biosynthesis, 11 in glycine, serine and threonine metabolism, 17 in valine, leucine and isoleucine biosynthesis and degradation, and 12 in cysteine and methionine metabolism.

#### (ii) Metabolism and biosynthesis of other amino acids

In addition to standard amino acid metabolism, 23 gene families were selected to target the metabolism of non-standard amino acids, which are not directly produced by cellular machinery, but formed by post-translational modification. The non-standard amino acids are generally essential for the function or regulation of proteins, such as better binding of Ca^2+^ [41]. Among the selected gene families, six were involved in selenocompound metabolism, four in D-glutamine and D-glutamate metabolism, three in cyanoamino acid metabolism, five in beta- and D-alanine metabolism, three in glutathione metabolism, and three in taurine and hypotaurine metabolism. A detailed list of gene families as well as involved non-standard amino acids can be found in Table S2.

#### (iii) Carbohydrate metabolism

Carbohydrates are critical nutrients for both human hosts and microbiota, and are also mediators that control the complex relationship between microbes and their human host [42], [43]. Only a limited portion of carbohydrates can be digested by human hosts, while the rest may be degraded by the gut microbiota [42]. Metagenome sequencing analysis has shown that the human gut microbiome contains a large number of genes related to carbohydrate degradation [24], [25]. We selected 35 gene families targeting central carbon metabolism (pentose phosphate pathway, TCA cycle, pyruvate, propanoate, and butanoate) and complex carbohydrate metabolism (starch, sucrose and pectin). Among these, six were selected for their important roles in pentose phosphate pathway, eight in pentose and glucuronate interconversions, four in pyruvate metabolism, four in propanoate metabolism, four in butanoate metabolism, six in starch and sucrose metabolism, four in fructose and mannose metabolism, and four in galactose metabolism,

#### (iv) Energy metabolism

Microorganisms are able to gain energy from multiple metabolic pathways, such as carbon fixation, methane metabolism, nitrogen metabolism and sulfur metabolism [44]. Fourteen gene families involved in energy metabolism were selected. Among these, three were selected for their important roles in methane metabolism, five in nitrogen metabolism, four in sulfur metabolism, and four in carbon fixation pathways.

#### (v) Glycan biosynthesis and metabolism

The human microbiota residing in the intestine play important roles in degrading glycans and polysaccharides, including dietary plants, animal-derived cartilage and tissue, and host mucus [45]. The polysaccharides synthesized by bacteria can also induce immune responses that are beneficial to bacteria, host, or both [46]. To monitor microbial related glycan metabolism processes, 14 gene families involved in lipopolysaccharide biosynthesis, peptidoglycan biosynthesis, and glycosaminoglycan degradation were selected. Among these, five were selected for their important roles in peptidoglycan biosynthesis, five in glycosaminoglycan degradation, two in lipopolysaccharide biosynthesis, and two in other glycan degradation.

#### (vi) Lipid metabolism and biosynthesis

Lipids are not only essential components of the human body, but also contribute to many pathological processes, such as obesity, diabetes, heart disease, and inflammation [47]. The biosynthesis and degradation of lipids could be carried out by both human cells and microbial communities. Previous studies have shown that microbial metabolism of lipids in the gut promotes atherosclerosis [48], [49]. Six key gene families involved in fatty acid metabolism (acetyl-CoA acyltransferase and beta-ketoacyl-acyl-carrier-protein synthase), glycerolipid metabolism (glycerol kinase), sphingolipid metabolism (beta-D-galactosidase), ketone bodies synthesis and degradation (butyryl CoA acetate CoA transferase), and bile acid biosynthesis (conjugated bile salt hydrolase) were selected.

#### (vii) Metabolism and biosynthesis of cofactors and vitamins

Cofactors are organic or inorganic non-protein chemical compound that are bound to and responsible for a protein’s activity. Organic cofactors are typically vitamins or are made from vitamins. A metagenomic study showed enriched vitamin and cofactor biosynthesis genes were observed in developing infant guts [50]. Also functional genomics analysis showed that some bacteria were unable to synthesize several vitamins, cofactors, and amino acids, and need to be taken up from the human intestine [51]. All these studies showed a complicated relationship between the host and its microbiota. Here 17 gene families involved in biosynthesis and metabolism of pantothenate, CoA, riboflavin, vitamin B6, thiamine, biotin, porphyrin, chlorophyll and folate were selected. For example, gene families encoding 3-demethylubiquinone-9 3-methyltransferase, riboflavin synthase, pyridoxal kinase, and thiamine kinase that function as the terminal step of biosynthesis of ubiquinone, riboflavin, thiamine, and vitamin B12 were selected, respectively.

#### (viii) Metabolism and biosynthesis of terpenoids and polyketides

Terpenoids and polyketides are natural products that can be found in all living organisms, with the potential function of anti-inflammatory and anticancer though the majority of them remain functionally unknown [52]. Five gene families related with terpenoid biosynthesis were selected.

#### (ix) Nucleotide metabolism and biosynthesis

Nucleotides are the basic structural units of DNA and RNA, and also participate in cellular signaling as well as cofactor synthesis. We selected 13 gene families involved in nucleotide metabolism.

#### (x) Translation

Three gene families involved in translation processes were selected.

### Summary of HuMiChip probes and target information

A total of 36,802 probes targeting 139 gene families were designed for HuMiChip, covering 50,007 coding sequences (CDS). Among these, 25,003 were sequence-specific probes that each probe targets only one sequence, and 11,799 were group-specific probes that each probe targets multiple sequences with high similarities (Table 1). Specifically, 15,175 (41.2%) probes targeted 59 amino acids metabolism genes, 6,217 (16.9%) targeted 23 genes for metabolism of other amino acids, 9,386 (25.5%) targeted 35 carbohydrate metabolism genes, 4,992 (13.6%) targeted 14 energy metabolism genes, 6,507 (17.7%) targeted 14 glycan biosynthesis and metabolism genes, 2,415 (6.6%) targeted 6 lipid metabolism genes, 3,660 (9.9%) targeted 17 cofactor and vitamin metabolism genes, 1,841 (5.0%) targeted 5 terpenoids and polyketides metabolism genes, 4,437 (12.1%) targeted 13 nucleotide metabolism genes, and 429 (1.2%) targeted 3 translation genes. Also, 80×8 16S rRNA gene degenerate probes as positive control, 3×563 negative control probes designed from seven thermophile strains, and 6000 identical common oligonucleotide reference standard (CORS) [53] probes for data normalization and comparisons were also included. Specificity for both positive and negative control probes as well as CORS was also verified by searching against NCBI nt database.

**Table 1:** Summary of designed probes and covered coding sequence information of HuMiChip based on gene categories.

Microbial functional process	#genes	#probes	#sequence-specific probes	#group-specific probes	#covered CDS
Amino acid metabolism and biosynthesis	59	15,175	10,245	4,930	21,241
Metabolism and biosynthesis of other amino acids	23	6,217	4,388	1,829	8,203
Carbohydrate metabolism	35	9,386	6,236	3,150	12,109
Energy metabolism	14	4,992	3,292	1,700	6,359
Glycan biosynthesis and metabolism	14	6,507	4,379	2,128	7,911
Lipid metabolism and biosynthesis	6	2,415	1,585	830	2,905
Metabolism and biosynthesis of cofactors and vitamins	17	3,660	2,464	1,196	4,879
Metabolism and biosynthesis of terpenoids and polyketides	5	1,841	1,247	594	2,517
Nucleotide metabolism	13	4,437	3,013	1,424	6,421
Translation	3	429	270	159	765
**Total** *	**139**	**36,802**	**25,003**	**11,799**	**50,007**

*Gene families targeting human microbiomes are selected from KEGG pathway database, and may participate in multiple pathways. The total number of probes and covered coding sequences is based on non-redundant genes included in all pathways, but it is not calculated as the sum of all sub-categories.

### Computational evaluation of probe specificity

**Figure 1:**
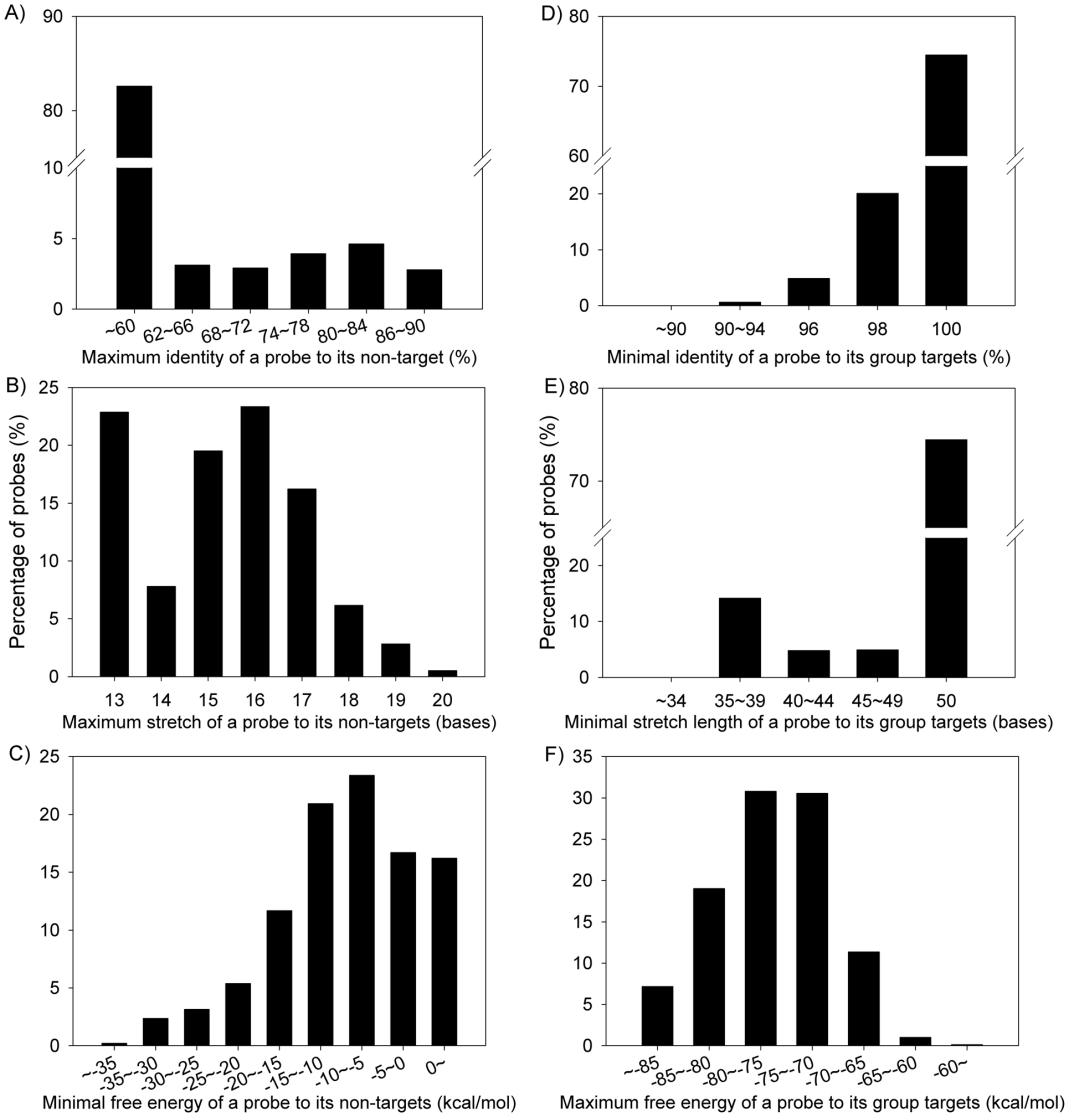
Computational evaluation of sequence-specific (A, B, C) and group-specific probes (D, E, F) at (A) maximal sequence identities, (B) maximal stretch length and (C) minimal free energy with their closest non-target sequences, and (D) minimal sequence identities, (E) minimal stretch length and (F) maximal free energy with their group targets.

The specificity for all HuMiChip probes was computationally evaluated against the MotherDB based on sequence identity, continuous stretch length, and free energy. For sequence-specific probes, the maximum identity, maximum stretch length, and minimal free energy to their closest non-target sequences were calculated. More than 83% of probes showed maximum sequence identities of 60% or lower to their non-targets. Only 7.4% of probes showed 80%∼90% sequence identity, 3.3% had 19∼20 base continuous stretch, and 5.5% had −35 to −25 kcal mol^−1^ free energy to their non-targets (Figure 1 A, B, C). For group-specific probes, the minimum identity, minimum stretch length, and maximum free energy to its group members were calculated. Approximately 75% of group-specific probes were identical to their group members, and more than 99% showed −85 to −65 kcal mol^−1^ free energy to their group members (Figure 1 D, E, F). All these results were consistent with the probe design criteria [26], suggesting the HuMiChip probes are specific to their targets.

### Application of HuMiChip to human gut and oral microbiomes

**Figure 2:**
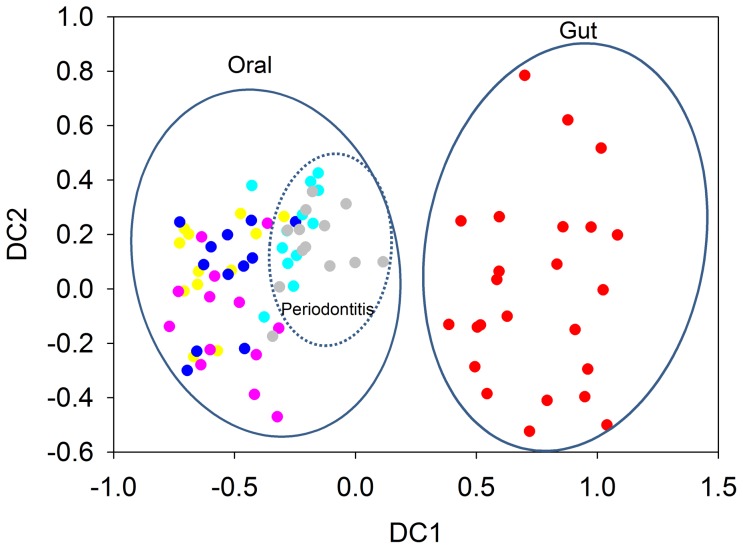
Detrended correspondence analysis of all functional genes detected by HuMiChip. A total of 86 samples were analyzed: 12 subgingival/supragingival plaque samples from healthy individuals (yellow), 25 supragingival plaque samples of which 12 from patients with moderate dental caries (blue) and 13 from patients with severe dental caries (pink), 25 subgingival plaque samples of which 12 from patients with moderate periodontitis (green) and 13 from patients with advanced periodontitis (gray), and 24 fecal samples representing human gut microbiome (red). A total of 14,460 probes detected in at least three out of 12 or 13 samples in each group were analyzed.

The HuMiChip was applied to analyze the functional composition and structure of human oral and gut microbiomes from 86 individuals (62 oral samples representing five groups of oral microbiota, and 24 fecal samples representing gut microbiota). Signal intensities for each probe were normalized by the mean signals from all spiked CORS probes. In total, 14,460 probes were detected in at least three out of 12 or 13 samples in each group, with an average of 6,699 probes detected per sample. Detrended correspondence analysis (DCA) of all detected genes showed that microbial communities in human gut samples were well separated from those in oral samples (Figure 2), suggesting significantly different microbial functional gene composition and structure between gut and oral microbiota. The significance was also verified by three different non-parametric multivariate statistical methods (ANOSIM: *R* = 0.707, P = 0.001; adonis: *F* = 0.29, P = 0.001; MRPP: δ = 0.365, P = 0.001). Also, a clear trend of separation of periodontitis patients’ oral samples from other oral samples could be observed (ANOSIM: *R* = 0.191, P = 0.001; adonis: *F* = 0.086, P = 0.001; MRPP: δ = 0.326, P = 0.001). No clear separation was observed between samples collected from healthy individuals and patients with moderate dental caries (ANOSIM: *R* = 0.046, P = 0.346; adonis: *F* = 0.011, P = 0.354; MRPP: δ = 0.297, P = 0.32) (Figure 2). However, significant differences were observed between patients with severe dental caries and individuals who were healthy or patients with moderate dental caries (ANOSIM: *R* = 0.186, P = 0.008; adonis: *F* = 0.074, P = 0.016; MRPP: δ = 0.332, P = 0.02), suggesting a progressive shift of microbial community composition and structure during the development of dental caries.

**Figure 3:**
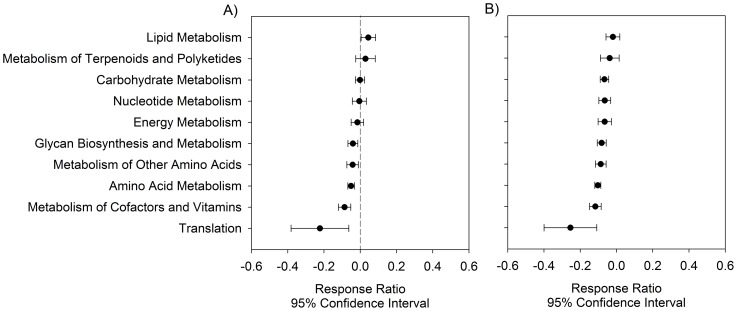
Response ratio analyses of changes of gene abundances based on categories. A) Moderate periodontitis patients vs. healthy individuals; B) Advanced periodontitis patients vs. healthy individuals. Error bar symbols plotted at the right of dashed line indicated increased relative abundances in moderate/advanced periodontitis patients, while error bar symbols plotted at the left of dashed line indicated decreased relative abundances in healthy individuals.

In order to see how oral microbiota changes at different stages of periodontitis, response ratio analysis of functional gene categories between moderate or advanced periodontitis patients and healthy individuals was carried out at a 95% confidence interval level. An obvious shift of most functional gene categories was observed between moderate and advanced periodontitis patients with most gene families having decreased abundances in advanced periodontitis (Figure 3A and 3B). For example, the abundance of lipid metabolism genes was significantly (P < 0.05) higher in moderate periodontitis patients compared to healthy individuals (Figure 3A), but became insignificant with decreased abundance in advanced periodontitis patients (Figure 3B). Also, no significant changes were found for gene categories such as carbohydrate metabolism, nucleotide metabolism, and energy metabolism in moderate periodontitis patients (Figure 3A), while significantly decreased abundances were observed in advanced periodontitis patients (Figure 3B). In addition, other gene categories, such as glycan biosynthesis and metabolism, metabolism of other amino acids, amino acid metabolism, metabolism of cofactors and vitamins, and translation, remained significantly decreased in both moderate and advanced periodontitis patients, but further decreased levels were observed in advanced patients (Figure 3A and 3B). All the above results indicated that a shift in oral microbiota with decreased abundances would be associated with the from-moderate-to-advanced periodontitis status, and HuMiChip is a useful tool for functional profiling of human microbiomes.

### Comparative evaluation of HuMiChip against NGS technologies

**Figure 4:**
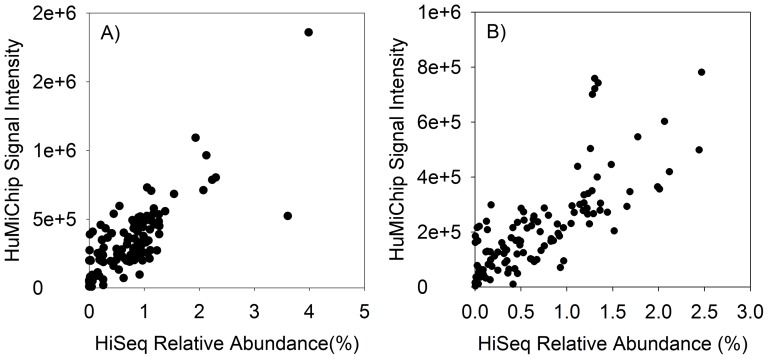
Comparative analysis of functional gene profiles as revealed by HuMiChip (total signal intensity) and NGS platforms (relative abundance): A) human gut samples. B) human oral samples.

The HuMiChip results targeting human gut and healthy oral samples were then compared with the relative abundances of corresponding gene families revealed by the HMP project using next generation sequencing (NGS). Gene family abundance datasets were downloaded from http://www.hmpdacc.org/HMMRC/, and profiles targeting human stool and subgingival plaque samples were extracted and analyzed. For the human gut samples, 121 of the 139 gene families showed a significant (P = 4.581E-027) correlation between HuMiChip and HiSeq analyses with a Pearson correlation coefficient of 0.79 (Figure 4A). For the human oral subgingival samples, 112 of 139 gene families had a significant (P = 2.033E-022) correlation with a Pearson correlation coefficient of 0.76 (Figure 4B). These results suggested that the gene family profiles identified by HuMiChip and NGS were well consistent with each other. In addition, it was noted that the lowest gene family abundance that could be detected by HuMiChip was about 0.001%, suggesting a high sensitivity of HuMiChip in detecting gene families of low abundance.

## Discussion

Microbial ecological microarrays such as GeoChip, PathoChip, StressChip, PhyloChip, HITChip, HuGChip, and several other microarrays have been developed and applied to analyze microbial communities in different habitats [18], [19], [20], [21], [22], [26], [54], [55], [56], [57]. These technologies were demonstrated to be powerful for functional and phylogenetic characterization of microbial communities, and linking them with ecosystem processes and functions. Most microbial ecological microarrays targeting human microbiomes are based on 16S rRNA genes, and are mainly suitable for phylogenetic profiling of human microbiomes. The HuMiChip developed in this study targeted 139 functional gene families that play important roles in various metabolic pathways, and can be used for functional profiling of these targeted gene families.

Since the HuMiChip developed in this study was developed mainly for microbial community analysis from different human body sites, specificity and sensitivity are two critical issues for successful application of microbial ecological microarrays. To insure the specificity of probes included in HuMiChip, previously experimentally evaluated parameters were used for highly specific probe design [58], [59]. In addition, extensive evaluations for functional gene arrays designed with the same criteria were carried out using pure culture DNA, mock community DNA, and environmental samples, suggesting high specificity and sensitivity for those microarrays [26], [36], [54], [60], [61], [62], [63]. Since the same criteria were used in the HuMiChip development, it is expected that the HuMiChip should have as high specificity and sensitivity as these functional gene arrays. Moreover, specificity for all probes were computationally checked and evaluated against the whole MotherDB, which included both full genomes and metagenomes. Finally, comparative evaluation of functional gene profiles revealed by HuMiChip and NGS technologies suggested significant correlations between these two approaches, and HuMiChip was able to detect functional gene families at as low as 0.001% relative abundance. All results suggest that HuMiChip is a specific and sensitive tool for functional profiling of human microbiomes.

The HuMiChip was applied to characterize the functional gene families in human gut and human oral microbiome. As expected, the overall structures of detected functional gene families in the human gut were clearly separated and significantly different from human oral samples, as suggested by both DCA and three non-parametric statistical methods, which was also consistent with several previous studies using NGS approaches of 16S rRNA genes and shotgun metagenomes [6], [64], [65]. Significantly different overall functional structures of oral microbial communities were also observed between healthy individuals and patients with periodontitis, indicating that periodontitis might be a disorder of the whole microbial community, which is generally consistent with previous studies [7], [8], [66], [67], [68]. Interestingly, significant differences were not observed between the oral microbiome from healthy individuals and patients with moderate dental caries, but observed between patients with severe dental caries and individuals who were healthy or with moderate dental caries. Such results suggested that the overall investigated functional gene profiles of microbial communities associated with moderate dental caries, which might be caused primarily by a few bacterial species such as *Streptococcus mutans* and *Lactobacilli* [9], were less affected. However, when dental caries develop to a severe stage, the whole microbial community was affected. Similar results were also observed between healthy individuals and patients with dental caries in a previous metagenomic study [6]. Both the changes of oral microbiome in patients with dental caries and periodontitis from moderate to severe status suggested a progressive change of functional gene profiles in response to the diseases. And HuMiChip successfully detected such progressive changes.

Periodontitis is a complex inflammatory disease in tooth supporting tissues, and is initiated by bacteria embedded in subgingival dental plaques involving complex interactions with their human hosts [67], [69]. The results revealed in this study provided some implications for the potential pathogenesis process of this human oral disease. For example, significantly increased abundances of functional genes involved in lipid metabolism were found in moderate periodontitis patients when compared with healthy individuals. Short-chain fatty acids can function to disrupt host defense systems using different mechanisms, such as the induction of apoptosis in immune cells [70], [71], [72] and gingival epithelial cells [73], and alteration of cell function and gene expression in human gingival fibroblasts [74], [75]. More interestingly, the abundances of lipid metabolism gene families decreased when periodontitis developed to an advanced stage, suggesting that lipid metabolism gene families might be important triggers for periodontitis development.

Currently, most functional profiling studies for human microbiomes were carried out by next generation sequencing (NGS) platforms, which should be used as gold standard for comprehensive analysis in exploratory studies of microbial communities. The HuMiChip developed in this study provides an alternative way for functional analysis of human microbiomes. Compared with NGS technologies, the main disadvantage for HuMiChip as well as other functional gene arrays is that the probes/genes covered by the chip are always limited, thus is not suitable for finding new genes/populations to define the extensive diversity of microbial communities in the environment. In addition, the limited coverage of probes/genes also restricts the accurate estimation of (relative) abundance in the community, making it more suitable for comparative studies but not exploratory studies. However, functional gene arrays still feature several advantages, especially for fast and cost-effective routine analysis of interested gene families. First, although sequencing technology is becoming cheaper and generates huge amounts of data, data analysis (e.g., assembly, function and taxonomy assignment) and interpretation is still extremely challenging and costly [76], [77], especially for complex microbial communities. In contrast, microarray data analysis methods are rapid, mature, and cost-effective. Second, NGS generates huge amounts of sequences (for both genes of interest or not), which is more suitable for discovery studies of both known and unknown gene content in the environment, while microarrays contain only genes of interest and can be used by researchers’ for routine studies of interested genes across many samples within a short time. In addition, due to the nature of NGS technologies, highly abundant gene families such as house-keeping genes are repeatedly sequenced, while low abundant, but functionally important genes are hardly sequenced, resulting in limited observations of these gene families. In contrast, gene families included on functional gene arrays are specifically selected according to researchers’ interests, and low abundant genes can be well captured. Thus, we recommend a complementary use of functional gene arrays for routine studies of interested gene families, and NGS for exploratory discovery studies of microbial communities. Novel gene sequences captured by NGS can be used for developing more comprehensive microarrays (e.g., functional gene arrays).

In conclusion, we have developed the HuMiChip for functional profiling of human microbiomes. A total of 36,802 probes targeting 139 gene families involved in key microbial functional processes in human microbiomes were included on HuMiChip, covering 50,007 CDS from 322 sequenced genomes as well as 31 shotgun metagenomes. Computational evaluation indicates that all HuMiChip probes are highly specific to their targets. Our analysis of the human oral and gut microbiomes suggests that the HuMiChip is a useful and high throughput tool to analyze the functional diversity, composition, structure, metabolic potential and dynamics of human microbiomes. The gene family profiles identified by HuMiChip were consistent with those obtained by NGS technologies. Further development of HuMiChip will target more sequenced genomes, as well as metagenomes, and develop strain/species-specific probes for strain/species identification[78].

## Supporting Information

Figure S1 **The pipeline for HuMiChip development.** Full microbial genome and metagenome sequences were collected as a MotherDB. Protein sequences were searched against seed sequences of selected functional genes using HMMER program. Corresponding nucleotide sequences of the HMMER confirmed sequences were extracted and subjected to probe designing by CommOligo. Specificity for the designed probes was evaluated against MotherDB. The best probes were then selected for microarray fabrication.
(DOCX)

Table S1 **Summary of functional gene categories, families, names, and their involved pathways and probe infromation on HuMiChip.**
(XLSX)

Table S2 **Gene families selected for non-standard amino acids metabolism.**
(XLSX)
