# Supplementary material for: Development of HuMiChip for Functional Profiling of Human Microbiomes
Source: PLoS One. 2014 Mar 4;9(3):e90546. doi: 10.1371/journal.pone.0090546 (PMC3942451; doi:10.1371/journal.pone.0090546)
Supplement: Figure S1 — The pipeline for HuMiChip development. Full microbial genome and metagenome sequences were collected as a MotherDB. Protein sequences were searched against seed sequences of selected functional genes using HMMER program. Corresponding nucleotide sequences of the HMMER confirmed sequences were extracted and subjected to probe designing by CommOligo. Specificity for the designed probes was evaluated against MotherDB. The best probes were then selected for microarray fabrication. (DOCX) [file pone.0090546.s001.docx]

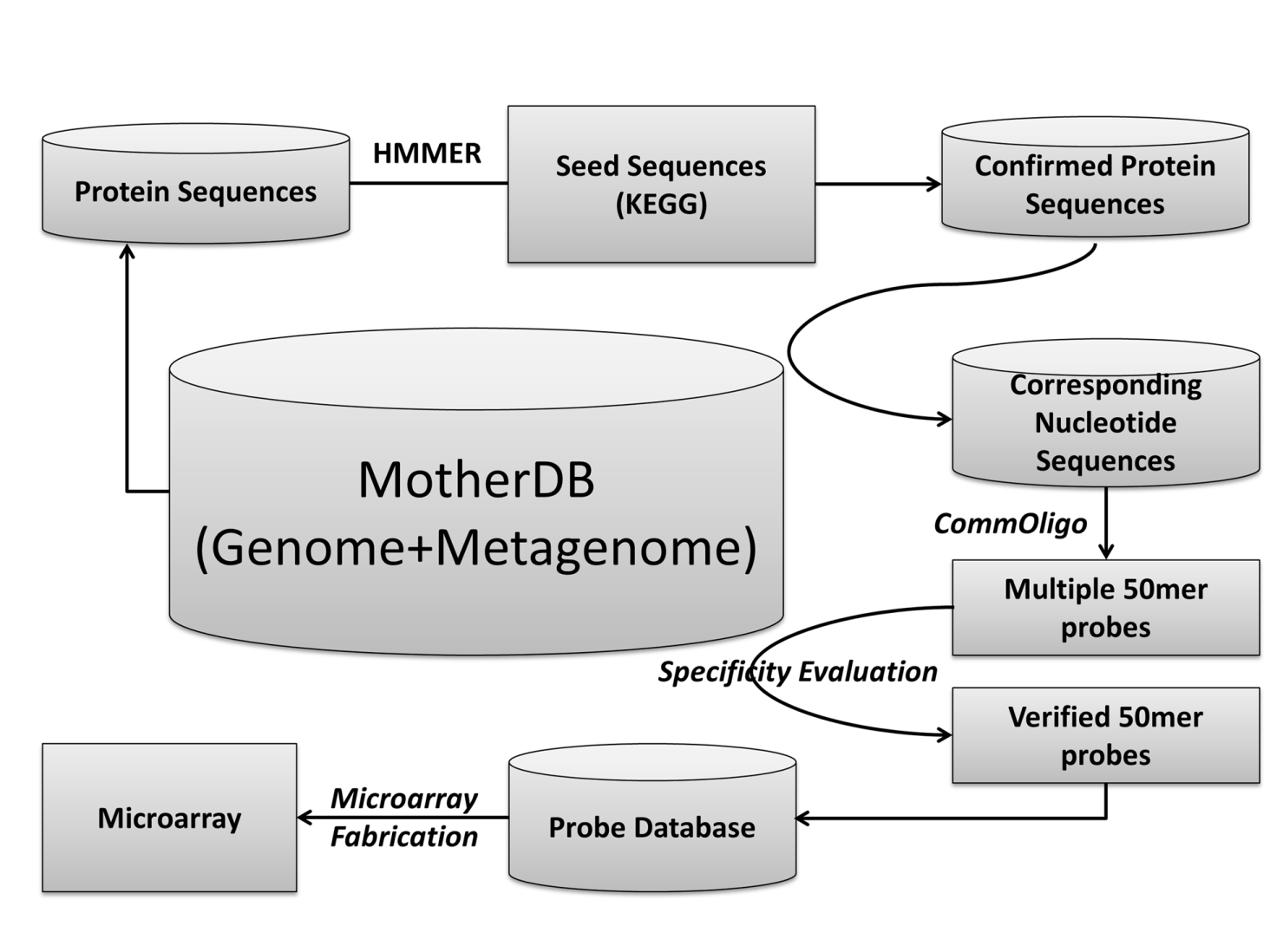


**Fig. S1.** The pipeline for HuMiChip development. Full microbial genome and metagenome sequences were collected as a MotherDB. Protein sequences were searched against seed sequences of selected functional genes using HMMER program. Corresponding nucleotide sequences of the HMMER confirmed sequences were extracted and subjected to probe designing by CommOligo. Specificity for the designed probes was evaluated against MotherDB. The best probes were then selected for microarray fabrication.
